# Poly‐aneuploid cancer cells promote evolvability, generating lethal cancer

**DOI:** 10.1111/eva.12929

**Published:** 2020-02-22

**Authors:** Kenneth J. Pienta, Emma U. Hammarlund, Robert Axelrod, Joel S. Brown, Sarah R. Amend

**Affiliations:** ^1^ The Brady Urological Institute Johns Hopkins School of Medicine Baltimore MD USA; ^2^ Nordic Center for Earth Evolution University of Southern Denmark Odense Denmark; ^3^ Translational Cancer Research Department of Laboratory Medicine Lund University Lund Sweden; ^4^ Gerald R. Ford School of Public Policy University of Michigan Ann Arbor MI USA; ^5^ Cancer Biology and Evolution Program and Department of Integrated Mathematical Oncology Moffitt Cancer Center Tampa FL USA

**Keywords:** cancer ecology, cancer lethality, cancer speciation, evolvability, metastasis, PGCC, poly‐aneuploid cancer cell, polyploid giant cancer cell, therapeutic resistance, therapy resistance

## Abstract

Cancer cells utilize the forces of natural selection to evolve evolvability allowing a constant supply of heritable variation that permits a cancer species to evolutionary track changing hazards and opportunities. Over time, the dynamic tumor ecosystem is exposed to extreme, catastrophic changes in the conditions of the tumor—natural (e.g., loss of blood supply) or imposed (therapeutic). While the nature of these catastrophes may be varied or unique, their common property may be to doom the current cancer phenotype unless it evolves rapidly. Poly‐aneuploid cancer cells (PACCs) may serve as efficient sources of heritable variation that allows cancer cells to evolve rapidly, speciate, evolutionarily track their environment, and most critically for patient outcome and survival, permit evolutionary rescue, therapy resistance, and metastasis. As a conditional evolutionary strategy, they permit the cancer cells to accelerate evolution under stress and slow down the generation of heritable variation when conditions are more favorable or when the cancer cells are closer to an evolutionary optimum. We hypothesize that they play a critical and outsized role in lethality by their increased capacity for invasion and motility, for enduring novel and stressful environments, and for generating heritable variation that can be dispensed to their 2N+ aneuploid progeny that make up the bulk of cancer cells within a tumor, providing population rescue in response to therapeutic stress. Targeting PACCs is essential to cancer therapy and patient cure—without the eradication of the resilient PACCs, cancer will recur in treated patients.

## 1 INTRODUCTION

All cancer would be curable if it were not for metastases and if metastases were not resistant to cancer therapies. These features result in the death of nearly 10 million people worldwide each year and close to 1,600 deaths every day in the USA (Bray et al., 2018; Siegel, Miller, & Jemal, 2018). These two properties—metastatic success and therapy resistance—occur because cancer cells are under selection to evolve traits that generate heritable variation: evolvability. That is, evolvability describes the capacity to evolve. Heritable variation is the fuel for natural selection. The rate of evolution in response to environmental circumstances is the product of heritable variation and the force of selection.

Cancer cells utilize the forces of natural selection to evolve evolvability. Evolvability describes a system where variation in phenotype is (a) heritable and (b) adaptive. The diverse cancer species inhabiting a patient initiate as a single monophyletic clade arising from a common ancestor in a speciation event where a lineage of normal cells goes from being part of the whole organism's fitness function to becoming its own self‐defined evolutionary fitness function (Table 1). Upon becoming the unit of selection, the initiating species of the cancer cell clade will be far from any evolutionary optimum. Being on the slope rather than the peak of its adaptive landscape means that traits conferring greater heritable variation will be favored. In addition, the heterogeneity of the emerging tumor can select for a diversity of cancer cells. A cancer cell with the capacity to generate heritable variation (evolvability) will be able to diversify more rapidly into different species that specialize on various aspects of tumor heterogeneity. A slower evolving species of cancer cells will be pre‐empted by the faster evolving species. Furthermore, any given region of a tumor is not static. There are constant changes in immune infiltration, blood flow, oxygen, pH, and toxic metabolite buildups (Amend & Pienta, 2015). Any evolutionary optimum, therefore, is constantly shifting, and the constant supply of heritable variation permits a cancer species to evolutionary track changing hazards and opportunities. Over time, the dynamic tumor ecosystem may also be exposed to extreme, catastrophic changes in the conditions of the tumor—natural (e.g., loss of blood supply) or imposed (therapeutic). While the nature of these catastrophes may be varied or unique, their common property may be to doom the current cancer phenotype unless it evolves rapidly to its dire circumstances. In the ecology of threatened species in nature, this is referred to as evolutionary rescue (Carlson, Cunningham, & Westley, 2014; Gomulkiewicz & Holt, 1995; Hammarlund, Von Stedingk, & Påhlman, 2018). While the details of a given catastrophe cannot be anticipated, evolving evolvability permits cancer species to adapt to the unexpected—and often catastrophic—temporal and special events.

**Table 1:** Evolutionary terms and definitions

Term	Definition
Species	A group of similar and related individuals defined by genetic separation and phenotypic differentiation from other species
Clade	A group of species that all evolved from a single ancestor
Evolvability	A biological system in which phenotypic variation is both heritable and adaptive; capable of adaptive evolution

Genetic instability, one of the hallmarks of cancer, enables cancer cells to evolve evolvability as an adaptation (Coffey, 1998; Cree & Charlton, 2017; Duesberg, Rausch, Rasnick, & Hehlmann, 1998; Hanahan & Weinberg, 2011; Heng et al., 2011; Loeb, Bielas, & Beckman, 2008; Maley et al., 2006; Mitelman, 2006; Tian, Olson, Whitacre, & Harding, 2010). There are several ways by which cancer cells can evolve to generate heritable variation that can be subjected to natural selection. Gene duplication permits mutation of one copy while retaining the original function of the other. Duplications permit the upregulation and amplification of useful metabolic processes. Conversely, gene deletions or epigenetic silencing by methylation can downregulate costly or unnecessary metabolic activities that are legacies of the normal cell's whole‐organism functions. Lax DNA repair mechanisms or other increases in mutation rates can contribute to greater heritable variation. Chromosomal rearrangements can change gene expression and gene regulation in ways that suppress or uncover new heritable variants. Increased demethylation of arbitrary or specific histones provides ways of creating heritable epigenetic variation that can cause single genes or suites of genes to be unmasked and expressed. Examples from other species suggest that chromosomal rearrangements that may significantly amplify heritable genetic variation could be more common in polyploid cancer cells (James, Usher, Campbell, & Bond, 2008; Selmecki et al., 2015; Yao, Carretero‐Paulet, & Van de Peer, 2019). The repeatable evolution of poly‐aneuploid forms of cancer species may be the primary culprit and the prime adaptation for cancers’ evolvability.

## 2 POLY‐ANEUPLOID CANCER CELLS AS A CONSTRAINT‐BREAKING ADAPTATION FOR STRESS RESISTANCE AND EVOLVABILITY

Multiple studies have described a minority population of physically large cancer cells within the tumors of patients with metastatic disease. This likely holds for most, if not all, cancer types that have the potential to result in therapeutically resistant metastases. These large cancer cells have unusually high genomic content (i.e., poly‐aneuploid) and exhibit remarkable resilience to stress (Amend et al., 2019; Brooks, Glogauer, & McCulloch, 2019; Chen et al., 2019; Erenpreisa & Cragg, 2013; Fei et al., 2015; Illidge, Cragg, Fringes, Olive, & Erenpreisa, 2000; Mirzayans, Andrais, & Murray, 2018; Niu, Mercado‐Uribe, & Liu, 2017; Zhang et al., 2014).

Virtually, all cancer cells are aneuploid (i.e., 2N+), containing an abnormal number of chromosomes or chromosomal fragments. The 2N+ cells display structural rearrangements, amplifications, and deletions. Polyploid describes increased DNA content of the cell's genome in its entirety. While polyploidy indicates complete sets of chromosomes, aneuploidy indicates varied copy numbers that may be specific for different chromosomes or chromosomal fragments within a single cell (Ben‐David & Amon, 2019). Thus, poly‐aneuploidy describes a whole‐genome increase in an aneuploid genome. Cancer cells with relative high genomic content generally occur at low frequencies and as poly‐aneuploids exist as 4N+, 6N+, or greater (Table 2).

**Table 2:** Genomic content terms and definitions

Diploid	Two complete sets of chromosomes; normal in a mammalian cell (2N)
Aneuploid	An abnormal number of chromosomes or chromosome fragments, including structural rearrangements, amplifications, and deletions (2N+)
Polyploid	One or more complete whole‐number duplications of the genome (e.g., 4N, 5N, 12N)
Poly‐aneuploid	One or more complete whole‐number duplications of an *aneuploid* genome (>4N+)

It seems that cancer species are able to exist in both 2N+ and poly‐aneuploid states, and that cancer cells of a clade shift between these states. As the poly‐aneuploids revert back to a 2N+ state, those that retain odd numbers of chromosomes or chromosome fragments might be more fit than those that do not. Thus, by the time the cancer is clinically detectable perhaps all of the observable cancer cells have cycled one or more times between poly‐aneuploid and 2N+ states.

We suggest that these poly‐aneuploid cancer cells (PACCs) (also referred to in the literature as polyploid giant cancer cells (PGCCs), multinucleated giant cancer cells, blastomere‐like cancer cells, osteoclast‐like cancer cells, and pleomorphic cancer cells) are a key adaptation that is repeated across virtually all patients with lethal cancer (Amend et al., 2019; Brooks et al., 2019; Chen et al., 2019; Fei et al., 2015; Illidge et al., 2000; Jinsong, 2019; Mirzayans et al., 2018; Mittal et al., 2017; Niculescu in‐press; Niu et al., 2017; Ogden, Rida, Knudsen, Kucuk, & Aneja, 2015; Zhang et al., 2014). We have adopted the naming convention of “poly‐aneuploid cancer cell” to emphasize (a) the distinction from normally polyploid cells/states (e.g., osteoclasts, blastomeres) derived from diploid progenitors, (b) the inclusion of both multinucleated and mononucleated PACCs, and (c) the aneuploid genome baseline of the polyploid genome.

We hypothesize that PACCs play a critical and outsized role in lethality by (a) their increased capacity for invasion and motility (high metastatic potential); (b) for enduring novel and stressful environments (successfully metastasize and be intrinsically therapy‐resistant); and (c) for generating heritable variation that can be dispensed to their 2N+ aneuploid progeny that make up the bulk of cancer cells within a tumor (providing population rescue) (Amend et al., 2019; Carlson et al., 2014; Lin et al., 2019).

**Figure 1:**
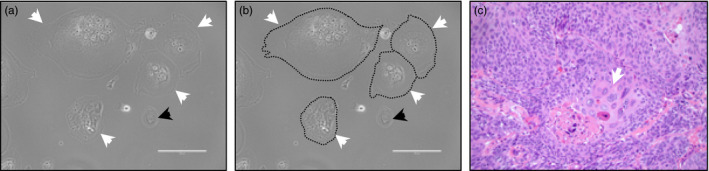
(a and b) Prostate cancer cell line PC3 treated with docetaxel for 6 days shows nearly all surviving cells are PACCs. (a) PACCs indicated by white arrow; non‐PACC indicated by black arrow; (b) shows an identical image with PACCs outlined for clarity (scale = 200 µM). (c) Invasive high grade papillary urothelial carcinoma H&E shows region of focal PACCs (indicated by white arrow)

Poly‐aneuploid cancer cells have been documented as emerging in response to stress, including therapeutic stress such as chemotherapy (Figure 1) (Amend et al., 2019; Lin et al., 2019, 2017). These data include tightly controlled in vitro assays as well as clinical data demonstrating increased PACC numbers in ovarian cancer patients following chemotherapy (Niu et al., 2017). Compellingly, published data also show that pressures present in the tumor microenvironment, such as low oxygen, induce the emergence of PACCs in vitro. PACCs are more common in metastatic lesions than in primary tumors, and they are more common in the primary tumors of patients with metastasis than those who have strictly localized disease (Fei et al., 2015). The presence of PACCs in the diagnostic specimen of prostate cancer predicts a dismal prognosis, with rapid disease progression and reduced overall survival (Alharbi, Marzo, Hicks, Lotan, & Epstein, 2018). Different functional events can produce PACCs including cell–cell fusion, endoreplication, and acytokinesis. It remains unclear whether one of these mechanisms predominates or whether they have different biological consequences (Amend et al., 2019; Chen et al., 2019; Illidge et al., 2000; Jinsong, 2019; Lin et al., 2019, 2017; Mirzayans et al., 2018).

Data suggest that PACCs survive dynamic environments (e.g., sudden onset of hypoxia or nutrient poverty) by exiting the cell cycle and entering quiescence or reversible therapy‐induced senescence, therefore protecting their genome and avoiding programmed cell death (Lopez‐Sanchez et al., 2014). The formation of a PACC phenotype is also associated with an increased capacity for motility and invasion. Motility allows PACCs to physically move into new environments, a rare feature among largely sessile epithelial cells (Fei et al., 2015). The quiescent state primes PACCs to survive the stress of circulation and colonization of a new metastatic site. Likewise, the quiescent PACCs survive anticancer therapy by protecting their DNA from damage and the subsequent activation of apoptotic programs. Once therapeutic stress is removed, as is the case with intermittent chemotherapy or tumor microenvironment hypoxia, the PACCs reenter the cell cycle and utilize their high genomic content to repopulate the tumor with nonpolyploid progeny. Reversion happens through a variety of potential mechanisms, including neosis (cell budding) and successful multipolar cell division (Amend et al., 2019; Chen et al., 2019; Illidge et al., 2000; Jinsong, 2019; Lin et al., 2019, 2017; Mirzayans et al., 2018). The progeny represents diverse heritable variants that propels further evolution of ecological niche filling and resistance.

Species of cancers within the tumor that can produce PACCs as a contingent strategy likely out‐survive those that do not. The capacity to form PACCs and then to return to non‐poly‐aneuploid states may be regarded as a key event occurring early in the cancer's phylogeny. Cancer cell species that can form PACCs replace those that cannot. Frequent occurrence of stochastic environmental stresses within a cancer population's microenvironment may provide the initial selection for creating PACC morphs. The PACC morph creates a resilient phenotype with several properties that are advantageous for responding to future stresses that any successful cancer species likely encounters over time. By providing a morph that provides robust resilience, PACCs predispose the cancer cell lineage for surviving and evolving to the unexpected. For example, being a circulating tumor cell that finds itself in a distant tissue and being exposed to anticancer therapy represent two unexpected and highly negative stressors for which PACCs may be cancer's solution. PACCs can provide the source of the “rescue effect” associated with the catastrophic event of therapeutic intervention (Carlson et al., 2014). The formation of PACCs, then, may represent the common convergent evolutionary event across patients that actuates metastasis and therapeutic resistance.

Poly‐aneuploid cancer cells provide a notable advantage over other mechanisms for generating heritable variation or evolvability (Amend et al., 2019; Brooks et al., 2019; Chen et al., 2019; Mirzayans et al., 2018, Vincent, Cohen, & Brown, 1993): PACC formation can be facultative. This means it can be modulated up and down as a contingent strategy. Increased mutation rates or reduced gene repair mechanisms can provide a steady supply of heritable variation. There may be contingencies when generating heritable variation at a high rate is disadvantageous (when traits are near evolutionary optima or when most genetic or epigenetic mutations are nonbeneficial) and when it is advantageous for other contingencies (evolutionary rescue or evolutionary tracking). As a conditional strategy, PACCs permit the cancer cells to accelerate evolution under stress and slow down the generation of heritable variation when conditions are more favorable or when the cancer cells are closer to an evolutionary optimum. That is, PACCs allow for facultative evolvability. In vitro experiments found that PACCs exist at a very low frequency within a cancer cell's population when the population experiences a consistent culturing regimen. Upon application of a lethal drug, PACCs increased in frequency to become the majority of cells (Lin et al., 2019). The higher mortality of 2N+ morphs partially explains some of the increase, but some of the increase comes from the rapid transformation of 2N+ cells into PACCs. With continued application of the drug, the PACCs produced the heritable variation needed for the emergence of fully resistant 2N+ cells (Amend et al., 2019; Lin et al., 2017). The now resistant population saw the proliferation of 2N+ morphs to repopulate the cancer species that then resumed a very low rate of transformation in PACCs. PACCs once again occurred at only low frequencies. Presumably over the evolutionary course of a cancer cell lineage, cells that produce PACCs facultatively have an advantage over those that have fixed rates of transforming cells into and out of PACCs.

## 3 PREPAREDNESS FOR THE UNEXPECTED IS CRITICAL TO THE EVOLUTION OF EVOLVABILITY

Inherited traits acquired through natural selection can be utilized for unexpected needs. For example, an organisms’ ability to hold its breath to prevent noxious toxins entering the lungs enables the otherwise unrelated trait of swimming underwater. The ability to form and then resolve PACCs may be a repeatable—and perhaps the most successful—adaptation for responding to strongly negative rare events that would otherwise be catastrophic for the cancer cell population. PACCs may allow the cancer cell species to win twice. First, by utilizing quiescence, PACCs are inherently more *resistant* to stressors than neighboring cycling 2N+ cells. Second, by utilizing their extra genomic content, PACCs *accelerate* the evolution of diverse solutions to these novel stressors. To our knowledge, no other form of genetic instability can provide both.

Cancer cell species that can produce PACCs, therefore, may represent an evolutionary archetype—an entity that has evolved the capacity for evolvability through increased, protected, and mobile genomic content. A central dogma of genetics reflects that the architecture of a genetic system simultaneously permits and constrains the heritable variation available to natural selection. A normal diploid cell, for example, is genetically and epigenetically programmed to perform a tissue‐specific set of tasks. A liver cell and a kidney cell each have the same genetic material but perform vastly different functions that are tightly regulated. Due to their increased and disordered genetic content, PACCs circumvent this architecture. PACCs evolve swiftly by providing heritable variation at a rate not available within a normal diploid cell (Yang et al., 2019). Similarly, utilizing aneuploidy to enable cross‐adaptation to therapeutic agents has been demonstrated in yeast, allowing rapid development of resistance in response to stress (Selmecki et al., 2015; Storchova et al., 2006). Natural selection can only respond to what has happened or is happening.

The certainty that a cancer cell will encounter rare circumstances is why natural selection can evolve evolvability as an adaption. Upon initiation, cancer cells are likely far from any evolutionary optimum. Natural selection will favor cells that evolve the most swiftly up their adaptive landscapes, toward the point where a cancer cell cannot improve one attribute without sacrificing another (defined as Pareto efficiency) (Henry, Hemery, & Francois, 2018; de Jong, 2007; Lloyd et al., 2016; Ng, Wang, Chowdhury, & Maranas, 2019; Schuech, Hoehfurtner, Smith, & Humphries, 2019). If uncertain catastrophic perturbations occur in a cancer cell's environment (or if the cancer cell unexpectedly finds itself in a completely foreign environment, e.g., metastasis), then it must evolve evolvability in response to the near certainty of catastrophic perturbations even if the exact nature or timing of a future perturbation is uncertain and unknown. Ironically, natural selection imbues most organisms with adaptations for dealing with the expectedness of the unexpected. This evolution of evolvability through PACCs has profound implications for understanding tumorigenesis and therapeutic resistance.

## 4 CONVERGENT EVOLUTION OF PACCS

The PACC phenotype represents a convergent adaptive response to stress. It appears to happen in all cancer cell lineages across all patients with metastatic disease. PACCs likely allow the cancer cells to produce gene duplications, repurpose redundant genes, generate novel variants from chromosomal rearrangements, and, perhaps most significantly, epigenetically access cellular programs typically restricted to subsets of tissue cell types, for example, macrophages, osteoclasts, and trophoblasts (Brooks et al., 2019; Diaz, Wood, Sibley, & Greenwood, 2014; Pereira et al., 2018; Yang et al., 2019). For example, PACCs may allow the cancer cells to utilize different metabolic strategies, movement characteristics, and stress response that is typical of these normal somatic polyploid cells. Such large multinucleated cells in normal tissues, found in humans as well as model animal systems (murine, *D. melanogaster*, and *C. elegans*), are nonproliferative and highly motile following the cell biology dogma of “differentiate versus proliferate”(Amini et al., 2015; Brooks et al., 2019; Diaz et al., 2014; Kim, Jin, Duan, & Chen, 2015; Pereira et al., 2018; Simionescu & Pavlath, 2011). These normal somatic polyploid cells, however, do not return to a 2N state, thus restricting their evolvability, a necessary constraint for maintaining total‐organism integrity and reducing malignant transformation of these cells.

Notably, PACC characteristics are not restricted to eukaryotic cells, are also observed in other organisms, and emerge as an adaptation to stress, providing evidence for ancestral genetic programs. Yeast, as noted above, exhibit a near equivalence of PACCs (Selmecki et al., 2015; Storchova et al., 2006). Some yeast form polyploids through meiosis without cytokinesis in response to toxins or adverse physical conditions. This permits rapid evolution of appropriate stress responses and later a return to a euploid state (Selmecki et al., 2015; Storchova et al., 2006; Yang et al., 2019). *Tetrahymena vorax*, a protist species of ciliate, exhibits two exemplars of stress‐contingent strategies (Gronlien, Hagen, & Sand, 2011). When food is generally unavailable, they can duplicate their genome to be essentially polyploid. When favorable times return, they reenter the cell cycle and can sustain several rounds of cell division without having to enter interphase and duplicate their DNA. It is unknown whether diverse heritable variants become possible as a result of this polyploid state. A number of protist species that exist in a haploid state will cease asexual reproduction and engage in sex by fusing into a 2N “polyploid” morph that subsequently undergoes meiosis to produce four 1N offspring. Similarly, some of invertebrate metazoans typically reproduce asexually and only engage in sex (via a “polyploid” state) when the environmental conditions become poor (e.g., water fleas, *Daphnia*) (Adamowicz, Gregory, Marinone, & Hebert, 2002; Vergilino, Markova, Ventura, Manca, & Dufresne, 2011; Xu et al., 2015). The ancient ameboid protist class Foraminifera alternates between haploid and polyploid states as a means of reproduction, even when conditions are completely devoid of oxygen (Akimoto, Hattori, Uematsu, & Kato, 2001; Pawlowski et al., 2003; Risgaard‐Petersen et al., 2006). The resulting propagules can be quiescent for years before starting to grow (Alve & Goldstein, 2010). Through passive suspension transport, these propagules have a remarkable ability to quickly colonize new habitats through opportunistic and pioneering species (Alve & Goldstein, 2003). Overall, versatile solutions to fluctuating ecological conditions are associated with the capacity for alternating genomic contents, resistance, and motility. PACCs seem to be recapitulating this adaptation that has been successful in so many free‐living unicellular species that have colonized virtually all places on Earth.

## 5 TARGETING THE EVOLVABLE EVOLVABILITY OF PACCS WILL BE NECESSARY TO CURE LETHAL CANCER

We see targeting PACCs as essential to cancer therapy and patient cure. Without the eradication of the resilient PACCs, cancer will recur in treated patients. One strategy to target these critical cells is to turn their capacity for evolvability into a fatal handicap. An evolutionary trap describes a situation in which an organism adopts an adaptive trait in response to an evolutionary environmental pressure that inadvertently makes it vulnerable to another environmental stressor (Basanta, Gatenby, & Anderson, 2012; Gatenby & Brown, 2018; Robertson, Rehage, & Sih, 2013). In essence, the organism is “tricked” into adopting a trait that will soon become extremely maladaptive. Known in evolutionary game theory as an evolutionary double‐bind, this strategy can be exploited to treat cancer cells that are otherwise resistant to conventional therapy combinations (Basanta et al., 2012; Gatenby, Zhang, & Brown, 2019; Zhang, Cunningham, Brown, & Gatenby, 2017). Effecting an evolutionary trap requires a two‐phased approach, with selection of the first agent or condition to promote a particular targetable adaptive response followed by an agent specifically selected to target the adaptive phenotype (Zhang et al., 2017).

**Figure 2:**
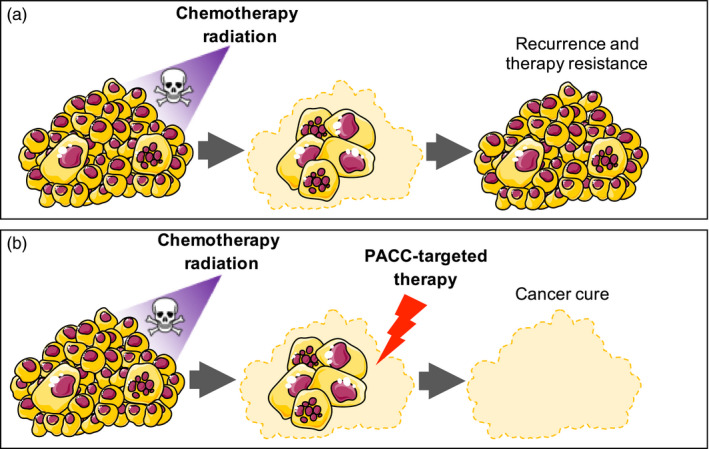
Using a current standard‐of‐care paradigm, systemic therapies including chemotherapy, radiation, and hormone therapy reduce overall tumor burden, but enrich for PACCs that then eventually give rise to resistant cancer cell population (a). Applying an evolutionary ecology strategy, using standard‐of‐care systemic therapy to enrich for PACCs and then directly targeting their peculiar vulnerabilities (e.g., requirement for centrosome clustering) or better drug delivery due to decreased tumor bulk opens the door for possible cancer cure in treated patients

The PACC cancer cell phenotype is defined by polyploidy, accompanied by an aberrant number of centrosomes (Amend et al., 2019; Chen et al., 2019; Illidge et al., 2000; Mirzayans et al., 2018). To divide evenly, normal cells possess two centrosomes, critical components of the microtubule organizing center (MTOC) that define the two poles of a dividing cell. Studies have shown that successful cell division of polyploid cells requires centrosome clustering to align mitotic poles and avoid multipolar division, mitotic catastrophe, and cell death (Antao, Marcet‐Ortega, Cifani, Kentsis, & Foley, 2019; Navarro‐Serer, Childers, Hermance, Mercadante, & Manning, 2019; Schatten & Ripple, 2018). Kinesin 14 (KIFC1) is essential to enable correct centrosome clustering and subsequent asymmetric cell division. Importantly, KIFC1 is dispensable in normal cells that do not require centrosome clustering, making it an ideal therapeutic target (Li et al., 2015; Sekino et al., 2019; Xiao et al., 2017). It may be possible to specifically target this process—only utilized in polyploid cells that undergo cell division—to specifically kill PACCs (Figure 2). To set the evolutionary trap, chemotherapy would be administered to expose the adaptive “evolvable evolvability” PACC morph (Basanta et al., 2012; Gatenby & Brown, 2018; Gatenby et al., 2019; Robertson, Ostfeld, & Keesing, 2017; Robertson et al., 2013; Zhang et al., 2017). The emerging PACCs would then be specifically targeted with a KIFC1 inhibitor to block centrosome clustering and lead to mitotic catastrophe when the cells attempt to divide.

## 6 SUMMARY

Poly‐aneuploid cancer cells species are isolated across time and space (i.e., different metastatic sites within the same patient), but utilize similar machinery to survive environmental and therapeutic stresses. Observations in cell culture as well as histologic tissues demonstrate a variety of PACCs with different genotypes and phenotypes within and among each community of cancer cells (Amend et al., 2019; Chen et al., 2019; Illidge et al., 2000; Lin et al., 2019, 2017; Mirzayans et al., 2018). Given that PACCs seem to exist across virtually all patients with lethal cancers, the mechanisms that underlie what appears to be a convergent survival mechanism across all cancer species must be defined. Just as there are many species of cancer cells, there are likely to be many species of cancer cells with the capacity to generate PACCs as a conditional state. These different species of PACCs, however, must share common genetic or epigenetic elements that allow the formation of the poly‐aneuploid evolvable “genotype/ phenotype” as a shared evolutionary adaptive response to stress. Ultimately, PACCs may explain the development of cancer lethality across individual patients. The versatility and role of PACCs in metastases and therapy resistance provide promising insights into possible new approaches to cancer therapy. Indeed, PACCs demonstrate characteristics such as genetic and geographic versatility that are key within the evolution of ecosystems in nature. Probing the characteristics of the PACCs as ecological strengths may provide novel ways to disrupt them and their role in rendering the metastatic disease lethal and ultimately untreatable.

## CONFLICT OF INTEREST

None declared.

## Data Availability

Data sharing is not applicable to this article as no new data were created or analyzed in this study.
